# Epidemiological review of confirmed Lassa fever cases during 2016–2018, in Plateau State, North Central Nigeria

**DOI:** 10.1371/journal.pgph.0000290

**Published:** 2022-06-24

**Authors:** Simji Gomerep, Martina Nuwan, Solomon Butswat, Joyce Bartekwa, Solomon Thliza, Christian Akude, Ayanfe Omololu, David Shwe, Rachel Reyna, Tomoko Makishima, Slobodan Paessler, Nathan Shehu

**Affiliations:** 1 Jos University Teaching Hospital Plateau State, Jos, Nigeria; 2 Plateau State Ministry of Health Nigeria, Jos, Nigeria; 3 John F. Kennedy Medical Center, Monrovia, Liberia; 4 Bingham University Teaching Hospital Plateau State, Jos, Nigeria; 5 Federal Medical Centre, Abeokuta, Ogun State, Nigeria; 6 University of Texas Medical Branch, Galveston, Texas, United States of America; Università degli Studi di Firenze: Universita degli Studi di Firenze, ITALY

## Abstract

Lassa fever (LF) is endemic in West Africa and constitutes a significant public health concern due to its potential for epidemics and associated high mortality. The first reported case and management of Lassa fever in Plateau State occurred more than 50 years ago. We set out to undertake a three-year epidemiological review of LF cases in Plateau State, North Central Nigeria. This is a retrospective study of all confirmed LF cases in Plateau State between 2016 and 2018. Plateau state Lassa fever- Line list and patient case records were used to extract relevant data. Lassa PCR was carried out at the NCDC accredited Laboratory network. Data analysis was done using STATA version SE14.1. Forty-four persons (44) had confirmed LF over the examined period, 18 (41%) in 2016, 15 (34%) in 2017 and 11 (25%) in 2018. The mean age was 29.7±14.6 years and 53% were males. Sixty-six percent (66%) of the patients resided in rural areas. It affected all local government areas (LGA) in the state except Pankshin, Jos East and Kanke LGAs. Twenty-five percent (25%) of the cases occurred among underprivileged communities of Jos North and another 25% in rural dwellers of Langtang North. Fifty-nine percent (59%) of cases occurred during the 1^st^ quarter, 27% the 2^nd^ quarter and 18% the 3^rd^ quarter of the year. The case fatality rate was 57%. LF is endemic in Plateau State. Prevention strategies must be sustained year round and target the youth, urban and rural underprivileged communities. There is also need for case management improvement to reduce mortality.

## Introduction

Lassa fever (LF) is an endemic viral haemorrhagic fever in Nigeria and across West Africa [1]. The first reported case of LF was in Lassa village, after which the disease was named, but the story of Lassa fever cannot be completed without mentioning that the first human case was brought from Lassa to Jos, Plateau State Nigeria. This was where the first case was brought for treatment, resulting in a subsequent nosocomial outbreak within the facility [2, 3]. Lassa fever constitutes a significant public health concern due to its potential for epidemics and associated high mortality. In addition, it also has the potential to be used for bioterrorism [4].

Lassa virus (LASV) is a single-stranded RNA virus belonging to the family *Arenaviridae*. It is a zoonotic disease whose reservoir is the multi-mammate rat of the genus *Mastomys* [1]. Clinical infection with LASV occurs primarily through exposure to food or household items contaminated with excreta or urine of infected rodents, processing of infected rats for consumption, droplet infection through the inhalation of tiny particles in the air contaminated with infected rodent excretions or reuse of infected needles. Person-to-person transmission occurs through contact with body fluids of infected persons, especially among healthcare workers, often due to a lack of appropriate infection, prevention and control (IPC) measures while providing care to hospitalized patients [5, 6].

The disease is mild or no observable symptoms in about 80% of infected people, but around 20% will develop a severe multisystem disease [5]. An estimated 300,000–500,000 cases and 5,000 related deaths occur annually across West Africa [7]. The actual incidence rate in Nigeria is unknown, but case fatality rates range from 3% to 42%, and over the last two years have remained between 20% and 25% [8].

In 2018, the Nigeria Centre for Disease Control (NCDC) reported a large number of cases in Nigeria, with over 600 confirmed cases and over 170 deaths. The reason for the increase is not thought to be due to any new virus strains, but may at least be partially explained by increasing surveillance capacities [8].

Historically, outbreaks occur during the dry season (November to April), although in recent years, cases have occurred also during the rainy season [9]. There is paucity of data on the magnitude of LF within Plateau State after over 50 years since the first identified case. This could affect the outbreak preparedness and resource allocation which would help control the disease. This study was therefore conducted to describe the epidemiology of LF, highlighting the magnitude of disease in a three-year period in Plateau state, Nigeria.

## Methods

### Study area

Plateau State has an area of about 26,899 square kilometers. It is the twelfth largest state of Nigeria by land mass, and is located in the North Central region of the country. It is geographically unique, as its boundaries are totally surrounded by the Jos Plateau covering the Central and Northern geo-political zones at an altitude of 1,200 meters (about 4000 feet) to a peak of 1,829 meters above sea level in the Shere Hills range near Jos. The Southern geo-political zone constitutes the lower plains. Plateau State has a population of around 3.5 million people, who are predominantly farmers. Plateau State has 17 local government areas (LGA) [10].

### Study design

This was a retrospective study of all confirmed LF cases in Plateau State between 2016 and 2018. The study population consisted of adult and children with suspected Lassa fever from urban and rural areas of Plateau state. The state capital and all LGA headquarters were defined as urban settlements, this was further classified as urban under privileged if the participant resides outside the planned city. While those residing outside state capital and LGAs headquarters were regarded as rural residents [11].

Data was retrieved between January and April 2019 from the Plateau State Lassa Fever Line list and case record form. [S1 Data]

A confirmed LF case was defined as a patient with suspected LF and confirmed by Real-Time Polymerase Chain Reaction (RT-PCR). Suspected LF cases were defined in accordance with the NCDC definition [12]. A patient with a fever ≥38°C for 3–21 days presenting with one or more of various symptoms (vomiting, diarrhea, sore throat, myalgia, generalized body weakness, abnormal bleeding, or abdominal pain), failure to respond to standard anti-malaria treatment and treatment for other common infectious causes of fever within 48–72 hours, as well as a history of recent contact with a probable or confirmed case of LF within 21 days of onset of fever, travel to regions of high risk of LF, or contact with body fluids or tissues of a deceased patient presenting with a febrile illness or signs and symptoms highly suggestive of LF. Plateau state Lassa fever Line list and patient case records were used to extract relevant data. The generated data was presented using descriptive statistics. STATA version SE14.1 software was used.

### Ethics statement

Plateau state Ministry of Health granted approval for the use of the secondary data and ethical approval was granted by the Jos University Teaching Hospital Institutional Review Board reference number: JUTH/DCS/IREC/127/XXXI/2422. All case data were de-identified before analysis for patient’s anonymity.

### Laboratory analysis

Lassa PCR was carried out at the NCDC accredited laboratory network using the same protocol. Blood samples were collected and viral RNA was extracted, purified and immediately used for RT-PCR. The LASV RT-PCR targeting the GPC gene was performed using QIAGEN One-Step RT-PCR Kit reagents as previously described [13, 14]. A primer set: S 36+ (5′- ACC GGG GATCCT AGG CAT TT-3′) and LVS_339-d− (5′-GTT CTT TGTGCA GGA MAG GGG CAT KGT CAT-3′), which targets a well conserved specific region within the Glycoprotein precursor protein (GPC) of the S RNA segment of the LASV was used. PCR products were separated and detected in a 1.5% agarose gel containing ethidium bromide and visualized by UV light. As a positive control, inactivated culture supernatant of cells infected with LASV strain isolated from cerebrospinal fluid was used.

## Results

**Fig 1:**
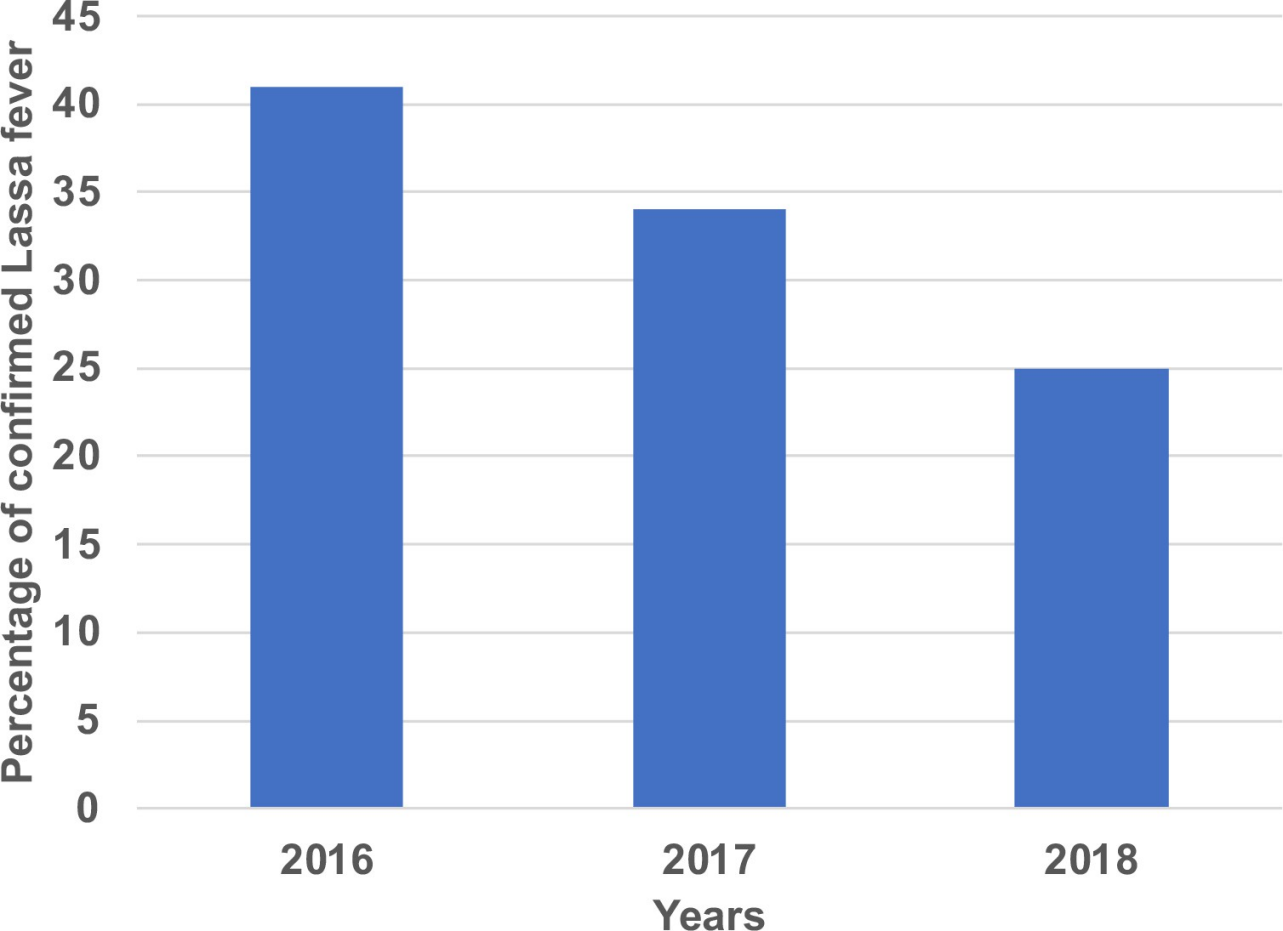
Distribution of confirmed Lassa fever cases by year of occurrence.

**Fig 2:**
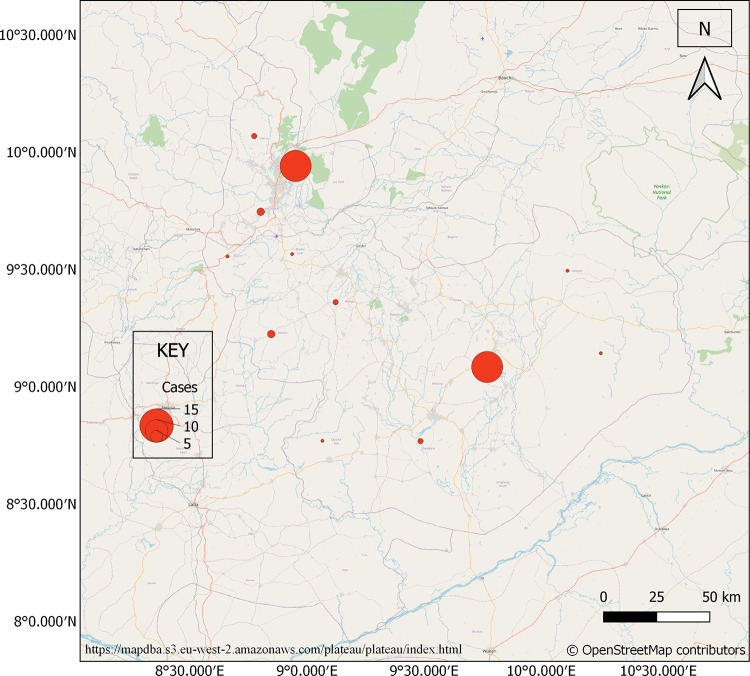
Distribution of confirmed Lassa fever cases across different local government areas of Plateau State 2016–2018. Base map from OpenStreetMap and OpenStreetMap Foundation [https://mapdba.s3.eu-west-2.amazonaws.com/plateau/plateau/index.html].

**Fig 3:**
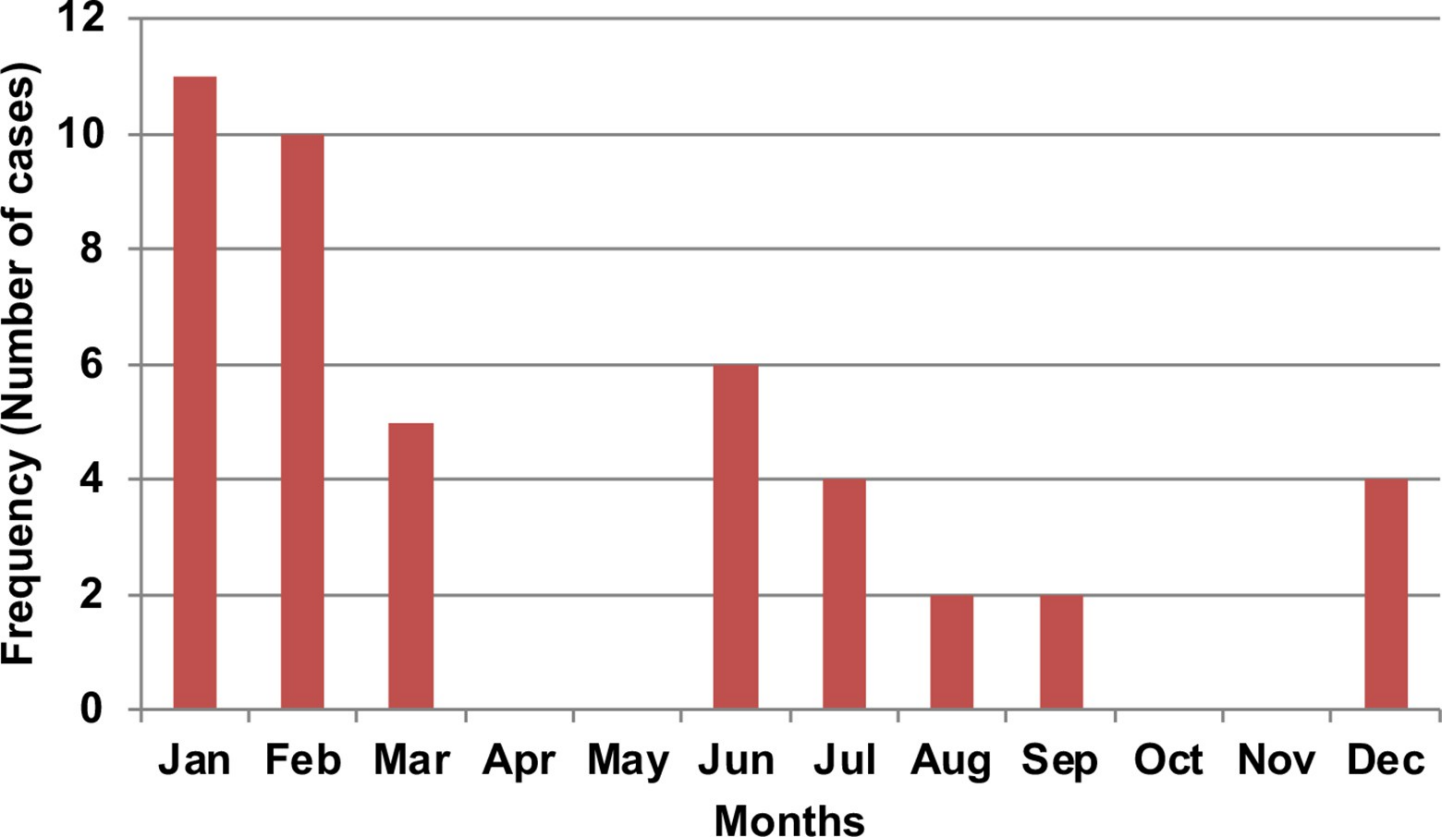
Distribution of confirmed Lassa fever cases in Plateau State according to month of presentation 2016–2018.

Forty-four persons (44) had confirmed LF over the examined period: 18 (41%) in 2016, 15 (34%) in 2017 and 11 (25%) in 2018, this is shown in **Fig 1**. The mean age was 29.7±14.6 years, and 53% were males. Sixty-six percent (66%) of the patients resided in rural areas. Cases were reported in all the local government areas (LGA) in the state except Pankshin, Jos East and Kanke LGAs (**Fig 2**). Twenty-five percent (25%) of the cases occurred among underprivileged communities of Jos North and another 25% in rural dwellers of Langtang North. Fifty-nine percent of (59%) of cases occurred during the 1^st^ quarter, 27% the 2^nd^ quarter and 18% the 3^rd^ quarter (**Fig 3**). The case fatality rate was 57%.

## Discussion

LF is endemic in Plateau State with a wide distribution in 14 LGAs out of 17 LGAs within the study period. The number of confirmed cases recorded may not accurately represent the real situation concerning all infections in Plateau State, as a large portion of infections may cause very little to no clinical symptoms. Consequently, patients may not present themselves to a healthcare facility for treatment. In addition, some may have been treated for other common endemic acute febrile illnesses like malaria or typhoid, without raising suspicion of LF [15].

Most of the patients resided in rural areas. Persons living in rural areas are known to be at a greater risk for contracting LF [16], which may be associated with their cultural practices and lower socioeconomic status. Practices such as hunting, preparing and eating rats as a source of protein and drying food items on the ground are common in these rural settings, which, coupled with a poor knowledge of risk factors for LF, may result in higher case numbers in these regions [15, 17, 18].

It is also interesting to note that two local governments constituted 50% of cases; urban underprivileged communities of Jos North and rural settlements of Langtang North LGA. Previous studies in Plateau State had indicated a higher burden of LF in Jos North LGA [19, 20]. Recent studies suggest that 80% of outbreaks are largely fuelled by independent zoonotic transmission events from infected rodent hosts [21]. These rural and urban under privileged settlements and slums are characterized by poor housing conditions and certain cultural practices that encourage the breeding and contact with the reservoir rodent, *Mastomys natalensis*.

The mean age of those infected indicates that the economically productive age group are the most affected by LF. This was also demonstrated in previous studies within Jos and in the neighbouring Bauchi state [19, 22]. The socioeconomic impact in the affected communities should be of great concern, especially in the rural and urban slums where most are farmers or manual labourers. LF occurred year round during the study period, with majority of the cases occurring in the first quarter of the year before the raining season. These may be attributable to increased human contact with the rodents as a result of activities such hunting of rodents, drying of food stuff in the open and bush burning that occurs at that time.

Historically, outbreaks occur during the dry season (November to April), although in recent years, cases have also occurred during the rainy season [8, 14, 23]. This has significant public health and policy implications to which clinicians and policy actors must pay attention, in order to maintain year-round preparedness and comprehensive outbreak control.

The mortality associated with Lassa fever in Plateau State is high. This may be due to delayed presentation, poor health seeking behaviour and other socioeconomic conditions such as the lack of accessible diagnostic testing for use within the community and difficulties in reaching health facilities [4]. Currently, there are only two functional LF treatment centres in Plateau State, both of which are located in Jos North LGA. These centres are not equipped to conduct laboratory confirmation tests and samples must be taken to distant reference laboratories outside the state, which further contributes to delayed treatment and poor patient outcome. The centres are also not adequately equipped to manage the complications associated with delayed presentation.

## Conclusions

This review has brought to bear, the facts that, Lassa fever infection is endemic in Plateau State, North-Central Nigeria and the role of seasonal peaks of the disease outbreaks observed predominantly among the agrarian rural and urban underprivileged communities in our setting. It is therefore recommended that year round health education to reduce risk of Lassa fever, widespread deployment of rapid diagnostic test kits for prompt diagnosis. This is needful to reduce the needless morbidity and mortality attributable to Lassa fever infections.

## Supporting information

S1 Data Raw data. (XLSX)
